# Client and Provider Perspectives on New HIV Prevention Tools for MSM in the Americas

**DOI:** 10.1371/journal.pone.0121044

**Published:** 2015-03-31

**Authors:** Sheri A. Lippman, Kimberly A. Koester, K. Rivet Amico, Javier R. Lama, Nilo Martinez Fernandes, Pedro Gonzales, Beatriz Grinsztejn, Al Liu, Susan Buchbinder, Beryl A. Koblin

**Affiliations:** 1 Center for AIDS Prevention Studies, Department of Medicine, University of California San Francisco, San Francisco, California, United States of America; 2 Center for Health, Intervention and Prevention, University of Connecticut, Storrs, Connecticut, United States of America; 3 Asociación Civil Impacta Salud y Educación, Lima, Peru; 4 HIV/AIDS Clinical Research Centre of the FIOCRUZ, Rio de Janeiro, Brazil; 5 Bridge HIV, San Francisco Department of Public Health, San Francisco, California, United States of America; 6 Laboratory of Infectious Disease Prevention, New York Blood Center, New York, New York, United States of America; David Geffen School of Medicine at UCLA, UNITED STATES

## Abstract

Men who have sex with men (MSM) in the Americas require targeted, combination HIV prevention approaches. We solicited client and provider perspectives on emerging prevention interventions including HIV pre-exposure prophylaxis (PrEP) and HIV self-tests through focus groups and in-depth interviews with 130 MSM and 41 providers across four sites: New York, San Francisco, Lima, and Rio de Janeiro. Among the MSM participants, we identified three prevention typologies: non-condom users, inconsistent condom users, and consistent condom users. Northern and Southern MSM differed in the variety of harm reduction strategies utilized: where U.S. MSM relied on condom use as well as disclosure and seroadaptive behaviors for prevention, condom use without disclosure or serostatus discussions was the norm in South America. Interest in new prevention technologies was shaped by the social context. U.S. MSM preferences differed by typology, such that non-condom users were interested in taking PrEP and using home HIV tests. MSM in Brazil, regardless of typology, were interested in exploring new prevention options. MSM in Peru demonstrated moderate interest but were less comfortable with adopting new strategies. MSM and providers’ opinions differed substantially with respect to new prevention options. Across sites, most providers were reticent to engage with new prevention options, though some NGO-based providers were more supportive of exploring new prevention tools. Both clients and providers will need to be engaged in developing integrated prevention strategies for MSM.

## Introduction

The incidence of HIV infection among gay and other men who have sex with men (MSM) continues to be high in many parts of the world, including the Americas [1, 2]. In the United States, HIV incidence among MSM is estimated at close to 3% per year [3, 4] and even higher among some sub-populations, such as young Black MSM [5]. In Peru, HIV incidence among MSM has been estimated to be at least 3% per year [6, 7] while in Brazil, HIV incidence among MSM has been estimated to be over 8% per year [8]. The epidemic in the Americas is concentrated among MSM, requiring targeted prevention efforts tailored to this vulnerable population.

The HIV prevention field has expanded significantly with new prevention strategies now available, including pre-exposure prophylaxis (PrEP) [9, 10], antiretroviral treatment as prevention [11] and approval of over-the-counter HIV self-testing kits [12]. While any one of these approaches may reduce HIV infection among MSM adopting them, it is the combination of these options that will likely produce an impact on the epidemic [13]. A number of questions remain about how these approaches should be optimized, including what combination may be most effective, how to target combination prevention strategies to particular groups, and how to tailor these options to maximize uptake among MSM. Furthermore, successful implementation depends on identifying and engaging with clinical and community-based providers who will deliver new biomedical strategies for MSM.

Optimizing combination HIV prevention requires understanding both client and provider perspectives of potential strategies. Whether prevention strategies are perceived as effective, useful and accessible depends on providers and client specific contexts and perspectives. Clients will not utilize HIV prevention tools if available options do not fit into their lifestyles and are not tailored to their needs; further, MSM have varied risk profiles and trajectories that may complement certain prevention strategies over others, and these needs may also change over time.

We conducted a qualitative study of MSM and prevention and care providers to identify current prevention strategies being used by MSM and to assess the acceptability and feasibility of implementing novel prevention interventions for MSM in four urban settings: New York City (NYC) and San Francisco, US; Lima, Peru; and Rio de Janeiro, Brazil. New prevention technologies were explored, including PrEP, rapid HIV self-tests, couples-based counseling and facilitated disclosure interventions, and web or mobile-based interventions [14, 15]. Main themes emerging from these discussions will inform the development and implementation of integrated prevention strategies for MSM in the Americas.

## Methods

Our multi-site qualitative study of MSM and health care and community based providers involved four study sites in North and South Americas: the San Francisco Department of Public Health (SFDPH), the New York Blood Center, the Asociación Civil Impacta Salud y Educación (IMPACTA) research clinic in Lima, and the Instituto de Pesquisa Clinica Evandro Chagas—IPEC/FIOCRUZ in Rio de Janeiro. The four sites are involved in a larger multi-site initiative, the Prevention Umbrella for MSM in the Americas (PUMA), which aims to determine an optimal mix of HIV prevention strategies for MSM in urban North and South American settings; selected sites represent cities with large HIV epidemics concentrated among MSM. Twelve focus group discussions (FGD) were conducted with MSM (averaging 6–7 participants per group; 3 per site) to assess perceived group norms and assess acceptability and feasibility of new HIV prevention interventions at a community level. Fifty-five in-depth interviews (IDIs) were then conducted with MSM (New York and San Francisco: 16; Lima: 9; Rio: 14) to follow-up on topics raised in FGDs that were better addressed in individual interviews, and to explore the influence of individual risk behaviors and situations on the acceptability of the interventions. FGD and IDIs used semi-structured interview guides to explore current prevention strategies, and opinions about pre-exposure prophylaxis (PrEP), HIV self-testing, couples-based interventions focused on counseling, and some were asked about mobile or internet-based technologies. The topic of web technology as a prevention tool was treated with significant variation between interview contexts and sites, emphasized as more of an educational tool in the South American sites, with some discussion of potential opportunities for web-based counseling in the FGDs. In the US sites, this topic was mostly addressed during FGDs, where MSM provided input on potential content and format for designing a sexual health website.

Following the qualitative data collection with MSM, forty-one key informant interviews (NYC: 7; SF: 9; Lima:12; Rio: 13) were conducted with health care providers, HIV prevention specialists working in non-governmental organizations, and decision-makers within public health departments (henceforth collectively referred to as “Providers”). Provider interviews elicited their perspectives regarding the usefulness and feasibility of implementing a combination of prevention components with MSM and explored where prevention services or strategies might be offered. Key informants were asked about their perceptions of prevention options described above. For the web or mobile-based prevention tool, providers were asked to provide feedback regarding a web-based tool that interactively generates a personalized sexual health promotion score based on answers to a series of behavioral questions. The web-based tool was developed in the interim year between client and provider interviews and as a result was explicitly included in provider interviews but only addressed in MSM interviews as more generic web-based tools.

FGDs and IDIs were conducted between August and November, 2011. Provider interviews were conducted between January and May, 2012. Of particular relevance, all interviews were conducted after the release of PrEP efficacy trial results from the iPrEx trial [10] but prior to the U.S Food and Drug Administration’s approval of the use of Truvada for pre-exposure prophylaxis and the OraQuick in-Home HIV Test Kits, both of which occurred in mid July 2012.

### Data collection

MSM were recruited to participate in IDIs and FGDs by peer educators and outreach workers at each site. In Peru and Brazil, recruiters visited venues frequented by MSM, including gay nightclubs, theaters, or recruited through social networks of the recruiters and other participants. In the US, recruiters utilized study advertisements placed at saunas, theaters, bars, clubs, or other places frequented by MSM. Eligibility criteria included self-identifying as a man who has sex with men, ages 18 or older, not known to be HIV-positive, and reporting at least 2 male anal sex partners in the prior 6 months. The MSM interviews were facilitated by outside consultants with expertise in qualitative research. In the South American sites, an English speaking consultant worked closely with a member of the local team (fluent in Spanish or Portuguese) to conduct MSM interviews. Interviews typically lasted 60–90 minutes, were audio-recorded and transcribed verbatim. MSM participants were reimbursed $50 in the US and $20 in Lima for their time; in Brazil, participants were reimbursed for transportation and snacks, in keeping with local regulations.

For provider recruitment, the study investigators identified a mix of local providers who could potentially deliver or influence the delivery of multicomponent HIV prevention tools at public clinics, in community based/non-governmental organizations serving MSM, at research sites, and in private practice. Recruited providers included counselors, nurses, and physicians, some of who were also program directors or administrators focused on HIV prevention and/or MSM. Providers were contacted by the local investigators to assess interest in participation and, upon a positive response, were contacted directly by the interviewers to schedule a meeting. Provider interviews were conducted by English, Spanish, and Portuguese speaking social scientists with extensive training in qualitative methods. Provider interviews in South America were conducted face to face in various locations, including a variety of offices and health care settings; provider interviews in New York and San Francisco were conducted over the telephone.

Ethical approval was received from the Committee for Human Research at the University of California, San Francisco; New York Blood Center Institutional Review Board; the Comitê de Ética em Pesquisa at the Instituto de Pesquisa Clínica Evandro Chagas (CEP/IPEC), Fiocruz, Brazil; and the IMPACTA Institutional Bioethics Committee in Lima, Peru. In New York and South America, participants were provided information about study objectives and procedures and written informed consent was obtained prior to interview. Participants in San Francisco were given a detailed information sheet about the study and verbal consent was obtained by asking each participant if they agreed to participate after reading the information sheet. As sites have differing local requirements for obtaining consent and providing incentives for participation, each IRB approved the local consent and reimbursement procedures for their site.

### Analysis

For IDIs and FGDs with MSM, the research team conducted the analysis in two phases. First, a deductive content analysis of both IDIs and FGDs was performed by the authors (led by KK) [16]. The team coded the IDI and FGD transcripts simultaneously to correspond to the domains of inquiry including: current HIV prevention practices, and attitudes about PrEP, HIV home testing, counseling approaches, and use of technology for prevention. Once coded, the data were examined and summarized within site and then compared across sites to derive patterns. Tables displaying site specific data were used to compare and contrast findings across sites. Presentations of the data were made to the local investigative team and site coordinators and vetted with the local community advisory boards.

Following the initial summarization of findings, the authors (SL, KK) conducted a deeper exploration of the IDI data with MSM participants in order to explore patterns of condom use that were noted during the initial analysis process; this led to categorizing participant narratives about personal HIV prevention strategies. Data was reduced into three prevention typologies which emerged from the data—classifying each participant into one group based on prevention attitudes and behaviors. The IDI data set was then analyzed through the lens of these typologies, assessing the reactions to prevention strategies and likelihood to uptake different prevention tools according to these typologies and across geographic sites. Matrices and tables were used to categorize and display data and to help understand the dimensions overall and across sites and typologies [17]. Following IDI data analysis, the authors re-read the focus group data to assess whether the findings from the IDI analysis resonated with the content of the FGDs; relevant quotes were pulled from both IDIs and FGDs. All participants included in this analysis identified as men; transgender women (n = 19) were interviewed at two of four research sites, but were not included in this analysis and are being considered separately.

The provider interviews were analyzed separately. The social scientists who conducted the interviews in each site summarized their findings in detailed reports organized by study objective and emergent themes. Findings from provider interviews across sites were compared and presented to the investigative teams and members of the community advisory groups for further interpretation and discussion.

## Results

Data were collected from 130 MSM and 41 providers, who participated in IDIs (n = 56 MSM; 41 providers) and FGDs (n = 74 MSM). MSM participants ranged from 18 to 51 years old, with an average age of 30 years. The MSM IDI analysis by typology included data from 55 participants (one participant was excluded as he revealed known HIV-positive status during the interview). Participation in each data collection component and MSM and provider profiles are detailed in Table 1 by site.

**Table 1:** Interview participants by research site.

**Participant characteristics:**	**Lima, Peru**	**Rio, Brazil**	**New York City, US**	**San Francisco, US**
**MSM**	**n = 27**	**n = 33**	**n = 36**	**n = 34**
IDIs	9^a^	14	16	16
FGDs (3 per site)	17	19	20	18
Average Age: years (range)	27 (18–37)	30 (19–50)	NA^b^	33 (24–51)
Calculated/mindful risk takers	2	3	2	4
Intimacy/pleasure seekers	5	5	4	4
Safety seekers	2	6	10	8
**Providers**	**n = 12**	**n = 13**	**n = 7**	**n = 9**
Role and institution	counselor (6); nurses (3); physicians(3)	counselor (6); social worker (2); physicians (3); outreach (1); NGO director (1)	public health administrators (2); physicians (4); NGO director (1)	counselor (3); advocate (1); NGO director (1); physicians (4)
	5 at NGOs; 7 in public system (of which 4 were associated with research centers)	5 at NGOs; 8 in public system (of which 2 were associated with research centers)	1 at NGO; 5 in public system; 1 in private practice (of which 3 were associated with research centers)	3 at NGOs; 2 private practice; 4 in public system (of which 4 were associated with research centers)

^a^ 10 MSM participants were interviewed; one participant reporting HIV positive status during the interview was excluded from analysis;

^b^ Demographic data not collected at New York site.

### Overall Approach to HIV Prevention & Prevention Typologies

Men across the four cities overwhelmingly cited condoms as their first line of defense against HIV. The majority of participants were quick to acknowledge the crucial importance and social desirability of condoms, regardless of personal use. However, the specifics of condom use, i.e., when, where, with whom, how often condoms were used, and how condoms were utilized in conjunction with other prevention strategies, differed by site. In Lima, men reported using condoms for oral *and* anal sex. In Rio, participants reported using condoms for anal sex, but typically not during oral sex. In New York, participants reported using condoms for anal sex, but definitely not during oral sex and often discussed serostatus with their sex partners as an additional component of enacting prevention. Finally, in San Francisco, participants reported using a combination of prevention strategies including condoms for anal but not oral sex, serostatus discussion with potential sex partners, and regular HIV testing. Men from New York and San Francisco described a broader and more nuanced repertoire of safer sex practices, including discussions of serostatus, while men in Peru and Rio were less comfortable with serostatus discussions and thus more consistently referred to condoms as the only foolproof prevention method.

Among the MSM participating in IDIs, we observed three consistent narratives related to different approaches to condom use that served to characterize men into one of three underlying groups: (1) the non-condom using group, or the *calculated or mindful risk takers*, included men who did not use condoms, were comfortable with their choice to pursue condomless sex, and typically reported making thoughtful decisions around partner selection and types of sex acts; (2) the inconsistent condom using group, or the *intimacy or pleasure seekers*, included men who aspired to use condoms consistently but also admitted to, and were often conflicted about, having specific criteria for condomless sex; and, finally, (3) men who always used condoms, or *safety seekers*, who were unwilling to have condomless sex under any conditions. These typologies are not mutually exclusive across all discourse, but do provide an approach to organizing highly complex behavioral decision making motivations and patterns that generally characterized a participant’s orientation towards sexual health protection. Of note, typologies themselves were aligned across the sites, however, there was some variation by site as to how each typology would approach the prevention options discussed, which is likely a result of differing availability of prevention options.

The participants who rarely used condoms (mindful risk takers) recognized the consequences in terms of risk for HIV acquisition. The majority of men in this classification felt that informed choices could keep them safer from HIV, though some of these men acknowledged that they remained HIV negative in the past by “pure luck.”

To be very honest only until recently, the only way I stay negative, I kind of think by pure luck. I have engaged in sex, gay sex for a long time. It's so funny because only recently I started using condoms. I'm really totally lucky.... about four or five months ago I got syphilis and I never caught syphilis before. And I caught syphilis through someone I see off and on, on a regular basis. And it was a shock that I caught it from them. You know what I'm saying? And really that has been a wakeup call…. (NYC, IDI 03)

The majority of these non-condom users were quite forthcoming about the risks they were taking as depicted by a participant from Rio:

These days, AIDS or whichever other STI, they can be treated or survived; that is not a justification for my actions, but it leaves me more reconciled… When I get tested, which I generally do every 6 months, I receive the result prepared for either a positive or negative result. If it’s positive—I know, I’m prepared for this. If it’s negative—great, I’m still negative. (Rio, IDI 12)

The second group of men discussed occasionally foregoing condom use, which typically happened when they were motivated by a desire for intimacy, greater pleasure or simply overwhelmed by passion. The HIV prevention narrative for “Intimacy and/or pleasure seekers” uniformly began with a firm proclamation that they “always used condoms”, however when asked whether there were *any* occasions when condoms were not used, they acknowledged instances of condomless sex. The normative pattern was to “always” use condoms, except with primary partners or trusted, regular sex partners or when decision making was compromised (e.g., alcohol use). Oftentimes, the pleasure of sex without a condom came at the cost of subsequent guilt and self-judgment as in the case of the second quote below.

…sometimes you know a person, you see he’s healthy, and one of my best friends, he gives you confidence, ‘let’s do it’ just like that, let’s be intimate without protection, … all of the sudden, the moment comes again, and you do it again, I don’t know what happens in the moment, but sometimes the desire of being intimate wins, it wins and you do it. (Lima, IDI 05)

Times when I haven't used condoms when it's anal sex, I think it's just kind of a heat of the moment thing and... it just feels better. And I hate to say that... you know it's not right even when you're doing it but you kinda just say, ‘Screw it,’ you know, and I mean and it's... it doesn't sound good even. I don’t... I'm not proud of saying that …. (SF, IDI 04)

The final and most common typology in the U.S., were consistent condom users collectively classified as “safety seekers.” This group of men fully integrated condoms into their sexual practices; they ‘insisted’ that they or their partners used condoms and reported feeling scared of any sexual activity without condoms. This group was distinguished by often seeking out multiple forms of prevention to guarantee they remained HIV negative.

So my strategy is, firstly, having a condom and secondly using it. And I already had relationships with HIV-positive partners knowing that they were seropositive, and I had no fear. We took all the precautions. (Rio, IDI 03)

I use condoms now for all my anal sex, top or bottom. I insist on it. It's... for me it's like carrying, you know, carrying keys with me out the door. Carrying my cell phone. If I'm gonna go out to hookup I'm gonna have condoms with me. It's... there's no discussion. (SF, IDI 12)

Well, condoms are a must. I don't care who you are, condoms are a must… and I will actually take people [to get tested] before we play. (SF, IDI 11)

Given these behaviorally distinct HIV prevention typologies, we assessed the perceptions about each of the prevention components by typology and site to explore when and with whom the prevention strategies could be most acceptable and successful in preventing new infections.

### Reactions to Prevention Strategies by Typologies and Site

The prevention strategies that appealed to participants were those that were consonant with their prevailing ideas about how HIV ought to be prevented, though patterns differed by study site. Generally, those who recognized that they could benefit from alternative prevention technologies (largely those not presently using condoms) were more enthusiastic about exploring ways the new technologies could bolster their prevention practices without necessitating condoms. Some who already felt they had a good handle on prevention (safety seekers) were less convinced that they required new and potentially less fool-proof options, though this was generally limited to safety seekers in the US. In the South American sites, interestingly, MSM’s interest in the prevention technologies depended on the location of the participant more than the typology of HIV-prevention approach. The majority of MSM in Rio voiced interest in all prevention options; MSM in Peru demonstrated cautious optimism about expanding prevention options, but more skeptical than their Brazilian counterparts; they were particularly reticent about any prevention strategy that might involve a potential for disclosure of one’s sexual orientation and HIV status.

#### PrEP

The majority of IDI participants from Lima were skeptical of using PrEP, though many did acknowledge that PrEP would be suitable for those most at risk, namely sex workers. The most adamant dismissal of PrEP was made out of concerns around the potential side effects (particularly weight gain) and the perception that it was not 100% effective. For some, they associated pill-taking with being sick and some thought daily use could be challenging; others said that pills would make members of their family curious (particularly bi-men) and would raise issues of confidentiality.

I'm not going to take a pill that a week later is going to give me a heart attack. (Lima, IDI 2)

It is notable that while the men participating in IDIs in Lima were more skeptical of PrEP than not, the men in the focus groups, particularly those who did not use condoms consistently seemed open to the idea. In one focus group most every participant thought PrEP sounded promising, particularly in combination with other prevention approaches. One man said:

It [the pill] gives you more confidence in yourself, you know. And to the person you are with, I mean, if I take my pill, I feel safer about having more sex with you, because I have more protection, plus the condom, plus circumcision, you feel safer about having sex with that person, it gives you more confidence. (Lima, FGD participant)

In Rio de Janeiro, almost all participants expressed interest in and willingness to take PrEP, both in the IDIs and the FGDs. Participants with more positive views on PrEP believed that it would alleviate underlying concerns about the risk of contracting HIV. Overwhelmingly, MSM in Rio considered PrEP to be an additional means of protection—a way to ensure they would not contract HIV. The idea of not using a condom while taking PrEP never spontaneously came up in discourse and when prompted, participants overwhelmingly perceived PrEP as an added value, not a replacement for condoms. This perception is consonant with the generally favorable culture of condoms. A participant who did not use condoms from Rio stated:

…in moments when I’m really turned on I let myself be convinced not to use a condom. This pill is another method [of prevention] that I can ingest—this would be an interesting option...” (Rio, IDI 12)

The typologies played a much larger role in New York and San Francisco, where MSM were split on the issue of PrEP. Generally, non-condom users demonstrated interest in PrEP as offering a form of “layered security.”

I would love to put my faith in a pill like that. That would be kinda cool. I'm, you know, I'm still, like, very sexually active right now. I'm not looking to settle down with someone in a monogamous relationship. So the idea of that [pill] happening would just, like, because so many times I just feel like when you're having sex there's just so much fear around it and, like, I would love to not have that fear… just the idea of that pill would just get rid of so much fear. (SF, FGD participant)

Disinterested men were largely ‘safety seekers’ and ‘intimacy/pleasure seekers’, generally comfortable with their current prevention strategies. In the US, those who had witnessed friends/ acquaintances endure side effects associated with early ARV regimens were often less amenable to the idea of taking PrEP. Some believed the drugs were somewhat toxic and hard on the body. Others raised issues related to the cost of the pill and the potential for risk compensation to occur. In San Francisco especially, many felt that PrEP was “great” for other people, but that they were not good candidates either because they were not pill-takers or because they already had solid HIV prevention practices in place. For these men, PrEP seemed best suited for the sexually “promiscuous or irresponsible” members of society (i.e., sex workers or barebackers).

#### HIV Home Testing

Following condoms, the most commonly reported prevention strategy across all sites was HIV testing, which some MSM reporting use for sexual decision making (i.e. sharing results of recent testing with potential partners) and others reported using to alleviate the regret attached to any episode of anal sex without a condom. An HIV negative test result absolved participants from feelings of guilt or anxiety.

By and large men across the four cities had a favorable reaction to the HIV self-test kit. Across typologies, MSM reported that HIV self-testing would provide privacy, alleviate shame and/or embarrassment, and circumvent the inconvenience of traveling to a clinic, waiting in line (a substantial obstacle in Rio), and feeling judged once there. Enthusiasm and support for the HIV self-test kits was, however, tempered by questions about the cost of the test and the threat of a false positive result. Additionally, while conceptually men liked the idea of HIV home testing, some were not interested in personally using a self-test, preferring to be tested with a counselor in a clinic. For those men that “loved” the idea because of the convenience factor, they thought the easy availability and convenience would lead them to more frequent testing and many suggested that they would use self-tests with a partner.

Some differences in attitudes towards HIV self-testing emerged between sites and by typology. In Lima, MSM were interested with some stating they would test more frequently if they could test in the privacy of their own home and most also agreed they would test with trusted, long-term partners, though some were concerned about giving up clinic-based counseling, which most identified as comfortable and appreciated. As with PrEP, men in Rio were keen to add self-test kits to the repertoire of prevention options and consistently referred to the benefits of convenience and privacy. The more tempered responses to home testing in Rio (and elsewhere) were usually in reference to concerns that some men would not be ready to handle testing alone. Only 1 participant in Rio put himself in this category, while others were confident they were ready for self-testing. The general consensus was very positive:

I think that anything that could facilitate our lives—I think it is welcome and this thing [self-test] would facilitate a great deal… I have a super busy life, and I think that most other people have a lot going on as well. So if you can do a test at your house in minutes instead of having to go out in traffic, lose a day, I think it would be really good… I also understand that in general some people would not be prepared to get a positive test result alone. (Rio, IDI 09)

Men in the US sites supported the idea of home testing, but about half preferred to continue to test in their customary locations, particularly those who were comfortable with their current testing routine. Typology did not heavily influence opinions on testing in any category except for the mindful risk takers who were personally interested in using the home test.

That’d be great. If it was affordable, I’d use it. There’s the convenience factor. It’s immediate. Privacy clearly would be an advantage there. (NY, IDI 06)

#### Couples Counseling as a Prevention Strategy

Men in South America were more amendable to the idea of couples-based interventions, most specifically counseling focused on improving communication with sexual partners. Most participants recognized couples counseling as beneficial, a way to build trust and encourage discussion about prevention, particularly as men in the South American sites reported that discussing HIV serostatus with sex partners prior to having sex was not at all normative. In fact, the very idea of asking for this conversation was anathema to most of the men interviewed, particularly in Lima.

The basic question is, ‘do you take care of yourself”? It is not, ‘have you taken any tests, or are you negative or positive’. (Lima, IDI 06)

Here no one asks [about serostatus]. What we have to do is use the tools that we have to keep ourselves safe. (Rio, IDI 02)

The reluctance to discuss serostatus was partially an issue of privacy and respect and also a way to avoid implicating oneself as a risk to potential partners by raising the question of status. Having a counselor to help negotiate or ease the conversation was appealing to most MSM in Lima with main partners; similarly, in Rio, all but one MSM with a main partner were very open to the idea of couples counseling.

At the U.S. sites, where disclosure is more common, less than half of the participants said they would seek couples counseling, and unlike the case with PrEP or home testing, this did not differ by prevention typology. About half of the participants in New York endorsed the idea of a sexual health counselor for couples, however, most participants felt they generally did not need sexual health counseling of any kind, and recommended counseling as a strategy for others in their communities (e.g., young gay men, couples early on in a relationship, gay men without access to social support).

People love to talk to people. Especially if they’re going to somebody who has more knowledge than them about a certain situation that they want to know. They can ask all the questions they want and the counselor can answer everything…and they can be open and honest. A lot of people just want to talk and get shit off their chest, you know? (NY, IDI 10)

In San Francisco, some men liked the idea of consulting a counselor to find ways to approach sensitive questions with partners. Other men in San Francisco expressed no interest in the concept of a sexual health counselor with a partner (or alone). Some stated that counseling is too stigmatizing, particularly within economically disadvantaged and ethnic minority communities, ineffective, or simply irrelevant.

There's a stigma with counseling, even educational counseling. There's a stigma to it. What are you doing wrong that you need it? (SF, IDI 10)

#### Using Technology to Facilitate HIV Prevention

Because discussions of technology were somewhat limited in the IDIs, there were insufficient data to explore perceptions of technology-based strategies by prevention typology; however, some important themes emerged in the data. Across the sites, all participants had experiences using the Web, but only a few described currently using it for the purposes of locating information about HIV and other sexual health related issues. In the U.S., participants described CDC-like websites as informative, but not user friendly. They would prefer a site with the same quality and trustworthiness of these sites, but in layman’s language.

In South America, some MSM mentioned using the internet to self-diagnose a sexually transmitted infection (STI) (e.g. if they had a wart or sore), but most used it solely for social networking and entertainment, mentioning high rates of utilization of sites like Orkut and Facebook, including use of these sites to identify sex partners. South American MSM generally liked the idea of using social networking sites for educational opportunities or the idea of creating a dedicated site for sexual health promotion that was interactive, easy to use, with accessible language, and MSM friendly.

In the US, topics that were spontaneously offered as recommendations by the focus group participants included access to counseling or sexual health experts on the web, ideally in real-time, and the use of vignettes (video clips) of real stories coming from people living with HIV to promote strategies around disclosure. Reception to the idea of “virtual” counseling was also well received in South American FGDs, however, participants were clear they would not want these virtual services to replace face-to-face counseling. Men in Peru stipulated that any virtual providers would need to be skilled in using basic and straightforward language to ensure client understanding, which they thought would be more challenging in a virtual setting.

### Providers’ Approach to HIV Prevention

Overall, providers across sites reported that a) they did broach the topic of HIV prevention with their clients/patients and b) their approach was open-ended and non-judgmental. Specifically, providers in the US sample described taking a patient-centered or sex positive approach to discussing HIV prevention with patients/clients. In most cases, providers described engaging in a dialogue with patients/clients rather than unilaterally delivering a one-size fits all directive to patients/clients to use condoms. Providers indicated that they felt comfortable when talking with patients about sexual behaviors and appeared to be well-informed about gay sex and sensitive to the “multi-factorial issues” that gay men face. In Brazil, the findings were similar to those in the US: providers reported they listened and worked together with their patients in a non-judgmental way to identify the most appropriate practices to reduce HIV risk within the social and cultural context. In Peru, providers emphasized the importance of establishing empathy and rapport, and stressing confidentiality with patients in order to facilitate prevention conversations. The general theme in Peru was to encourage patients/clients to reflect on their sexual risk taking.

Across sites, understanding of risk factors and forms of promoting risk reduction were closely tied to the providers’ experiences working with the MSM community. Providers from NGO services and those that worked closely and consistently with NGOs tended to express more understanding of the broader social and cultural contexts which influence MSM’s ability to practice safer sex behaviors. NGO providers were clear that this meant avoiding exclusive focus on condom use, as this for many of their patients was difficult—especially in long-term relationships. In contrast, some providers in medical settings had a tendency to emphasize condom use only. For some community-based providers, they expressed concern that colleagues in medical settings would need more training and education to be able to implement prevention strategies that require more dialogue and listening with MSM patients than they are accustomed to providing. Conversely, medical providers were unclear on how community-based providers would promote and deliver a biomedical prevention strategy (PrEP).

#### Providers’ Perceptions of PrEP

Across sites, the providers were conflicted about the potential for diverting resources used for treating HIV to cover the costs of PrEP. That said, U.S. providers were interested in PrEP, seeking further information, and conducting research on PrEP. Enthusiasm was dampened by what they perceived as a lack of demand and the potential barrier of prohibitive costs.

People are HIV positive and cannot get meds for free. It [PrEP] feels very unethical to me. It’s an issue for me. (SF, NGO-based provider)

We have a protocol at our center. We offer it. It’s one more thing to offer. It’s not been very successful. Patients are uninsured and therefore have no access to medications. Realistically cost is a barrier… (NYC, MD)

In Brazil and Peru, only providers from the research sites (where HIV prevention and treatment clinical trials have been conducted) were informed about PrEP. In both South American sites providers were unsure whether PrEP would be authorized or even considered as a national strategy, given the prohibitive costs and lack of support from the Ministries of Health (as of 2012, which has since changed in Brazil). Peruvian providers were hypothetically and cautiously optimistic about the potential of PrEP, though they also had reservations about the populations’ ability and interest in adhering to a regimen when not ill. Brazilian providers were also worried about poor adherence as well as risk compensation, but interested in thinking about how PrEP could be used to enhance prevention in combination with counseling and cautiously distributed in through the public health system.

#### Providers’ Perceptions on HIV Home Testing

Home testing drew the most inconsistent and emotionally-charged responses from providers across sites, where a continuum of opinions emerged, ranging from strong reservations to cautious support and interest. Those who were enthusiastic voiced strong support to expand access to testing and saw home test kits as an opportunity to let MSM make their own testing choices—whether they use home tests as their primary testing source or as an “in-between” clinic visit strategy. Those who were most reserved voiced concern about the preparedness of their clients for receiving an HIV positive result alone and handling the news if positive.

I am completely against it [HIV testing at home]…I have worked with HIV for more than 10 years, and each person reacts in a different way. People you think are going to completely fall apart end up receiving the news calmly and concerned about what they can do in the future. Others who you think are going to be completely fine end up falling apart in front of you, cry, think that their life is over…I think that without any form of counseling, it simply should not be done… (Rio, Infectious disease specialist)

There were also major qualms about how MSM would be linked into follow-up testing and care after receiving a positive HIV self-test result, and about interpreting and acting on a negative test result in light of the window period if not properly counseled. Other logistical concerns surfaced around structural barriers, such as laws stating a requirement for pre-test counseling as part of national HIV testing services (Lima) and whether restrictions for sale or distribution of home test kits would exist for populations under the age of 18. Some of those providers who did not like the idea generally, however, were open to the idea of providing home tests following an assessment of “readiness” or for repeat testers or clients in sero-discordant relationships.

#### Provider’s Perceptions of Couples Counseling

Providers interviewed were generally supportive of couples HIV testing and counseling across sites, recognizing a need in the community while also noting logistical stumbling blocks and a need to systematize the practice and a roadmap for implementation. The major concern was volume and human resources. If a counselor needed to meet with each member of the couple first and then together, introducing this into already overstretched clinics would strain resources. Providers were also puzzled about how one would determine who qualified as a couple, when this prevention approach would be most optimal, and whether counseling would only be HIV focused or include broader mental health and communication issues. In Brazil, the inclusion of couples counseling was perceived as politically important for recognizing gay and trans couples. In New York, providers were more apt to support this strategy for younger men in need of more support but also recognized that this service might not be attractive for young men. Interestingly, a large number of providers reported already providing this service informally—but none had a “partners’ program” per se, with guidelines or specific training. This is well illustrated in a quote from a medical provider in New York:

I do that [couples counseling] all the time, because every time somebody is in a new relationship, I know that getting rid of condoms is a goal and people are in a real rush to get there and so I very much talk about what I see as the components about making that decision. … We don’t make heterosexual couples use condoms in long term relationships, there’s no reason to make gay men in monogamous couples do that, but how do you negotiate that question? … Basically, I ask people are you willing, can you make an agreement that if this person comes to you I played around and we have to use condoms for 6 months. Do you agree not to go running screaming for the door, because without that level of commitment, then you’re not ready to give up condoms. People’s eyes open up. They never thought about it that way. I think it makes a big difference. … It would be great to have some professional advice on what I’m doing. (NY, medical provider)

#### Technology

All providers interviewed used technology in some ways to reach patients or clients either through websites or Facebook, YouTube or Twitter. The CBOs/NGOs maximized the use of social media to reach MSM. Providers agreed that in today’s world, it is necessary to use online tools for prevention and disseminating information, yet were hesitant about the extent to which these technologies could be forums for discussion in the absence of an educated moderator. For example, for those with experience with chat rooms, they noted that when they tried to insert prevention messages into sex chats, they were largely unsuccessful as they perceived people as not wanting to talk about prevention in these spaces.

The idea of “virtual” counseling or a web-based tool (having clients respond to interactive questions that generate a personalized risk score) got mixed reviews among U.S. providers. It appealed to some, but others were concerned about the veracity of the data provided and thus of the ensuing score. There was no consensus about the best format to complete risk questions or to present risk score results to clients (i.e. use alone or with a counselor).

I like the idea of people thinking about risk. The basic concept—I like it. But I’m concerned people will put in inaccurate information, not intentionally, underestimate risk and end up with an inaccurate summary because of that. The score is only as good as the information going in. (SF, Sexual health clinic rep)

Great strategy, but not alone, needs to be done with more. Good to have the support for a larger prevention strategy but not as a stand-alone thing. (NY, CBO provider)

However, providers were more encouraging in Peru and Brazil that such a tool could both be helpful to MSM using it alone and definitely as a tool to facilitate prevention conversation and support (but not replace) counseling.

Good idea to have a patient score that could be like a speedometer, little risk is green, yellow, red. It would be a great tool. (Lima, Counselor)

## Discussion

We explored current approaches to HIV prevention, as well as perceptions of new prevention options among MSM and health providers in the Americas in order to assess which prevention options might be delivered and by whom. Three main typologies of HIV prevention orientations emerged: non-condom users or mindful risk takers, inconsistent condom users or intimacy/pleasure seekers, and consistent condom users or safety seekers. These typologies may play an important role in determining who may be interested in and benefit from new prevention technologies. Quantitative studies have sought to create typologies based on reported behaviors, similar to those described in the current study [18, 19], with particular attention on how risk trajectories can inform PrEP allocation [18]. Analysis of multiple prevention strategies by site and typology may guide future delivery of combination prevention for MSM. In the U.S., participants reporting less condom use were most interested in taking PrEP and using HIV home test kits—and are likely the best candidates and early adopters of these new prevention tools. Notably, these men also reported behaviors that likely placed them at highest risk for HIV, appeared to be aware of this vulnerability, and were interested in accessing new prevention technologies.

We observed that MSM in Rio, regardless of their condom use profile, were interested in expanding prevention options, including PrEP, self- or home-testing, couples-based counseling, and technology-based prevention, all of which were popular. The finding that participants in Rio universally support availability of all options is consistent with Brazil’s historical socio-political movements around public health and access to HIV/AIDS care; there is an acute awareness that health is a right that grew out of the grass-roots struggle for a universal care system [20–22]. At the same time, the National AIDS Program (NAP) has reinforced this activism by instituting policies to secure lower prices of ARVs, including compulsory licensing and domestic production of patented generics [22, 23]. With that backdrop, if the public sector can continue to support program expansion, there is clear client support for moving forward with expanded prevention options in Rio. In contrast, the men in Peru were moderately interested in the new prevention options. While a handful of participants had tried new strategies as part of scientific research (some had participated in the iPrEx trial), they did not appear to approach the issue of prevention from an expectation of change. Interest in new technologies in Peru was informed by a decade of prevention limited to condom use and testing programs and participant responses seemed tempered by fear of losing access to current prevention. This more hesitant orientation aligns with current assessments of Peru’s national HIV/AIDS program, which, despite adopting human rights discourse and recognition of marginalized groups as partners in HIV programming [24], has remained largely condom focused since its inception to the exclusion of other prevention tools for MSM [25].

A striking difference between the Northern and Southern sites was the variety of HIV prevention strategies described by men in North America, where men spoke of sharing information with sex partners, sero-status discussions, as well as testing regularly. Our observation that prevention-related decision making and harm reduction strategies based on sero-status was occurring in U.S. cities has been established previously [19, 26–28]. As is consistent with the available literature from South American MSM [29], very few MSM reported serostatus discussions and there was little evidence that knowledge of serostatus is sought for sexual decision making or sexual positioning choices (seroadaptation). Instead, in South America, stigma and tension around disclosure precluded candid discussion and led men to rely heavily on condoms and/or to indirectly pursue safer sex by seeking partners in ‘safe’ environments (e.g. MSM leaving testing venues).

We noted a divide between MSM and providers’ opinions with respect to the acceptability, feasibility, and utility of many of the prevention strategies we explored. For example, providers raised the issue of risk compensation with use of PrEP across sites, and yet, the potential consumers talked explicitly about the addition (not a replacement) of PrEP to existing strategies. Despite the fact that fears of risk compensation have not come to fruition to date in research on PrEP [30, 31] or in circumcision trials [32], providers continue to express concern about provision of new methods and the potential impact on condom use [33]. Perhaps the most salient example of the provider/client division was the differing attitudes about home testing in South America, most markedly in Brazil. Providers there and elsewhere expressed concern about their clients’ understanding test results (and the window period) and clients’ inability to cope with potentially stressful test results. Most MSM agreed that not everyone would be equipped to use home tests, but few saw themselves in this light. In fact, some MSM stated that those who were ill equipped would not seek out self-testing anyway. Acceptability and feasibility studies of home testing in the US, Brazil, and elsewhere have demonstrated that MSM are eager to access this technology [34–38]. It is possible that home tests elicited particularly cold reception among providers because they challenge long-held beliefs: home testing implies that HIV diagnosis and monitoring can become a user-based technology, like glucose monitoring, which could challenge AIDS exceptionalism [39]. Taking testing out of the clinic may also seem contrary to the long fought battle to make clinics friendlier and more sensitive to the needs of MSM. We believe both improved clinic-based and self-testing options are needed to meet the variety of testing preferences among MSM across sites.

It is also notable that while providers were confident in their ability to engage patients in an open-ended and non-judgmental dialogue around prevention, their cautious approach to thinking about newer strategies (e.g. PrEP and self- testing) may indicate reservations about their clients’ ability to engage in determining their own prevention strategies—particularly in Southern sites where providers-client relationships are historically hierarchical. Openness to new technologies was more evident in CBO or NGO settings and research institutions. However, if MSM are offered combination prevention options through local health departments, the public providers could prove hesitant partners in implementation. If PrEP implementation and public distribution of self-tests move forward, it will require steps to sensitize providers to more closely align to the needs and preferences of MSM they serve [33]. Providers will also need detailed local guidelines to facilitate the delivery of new prevention approaches. For example, even where couples testing and counseling is implemented, providers stated a need for guidance, which could come in the form of counseling guidelines or expand into digital tools [40].

These qualitative data are not designed to be generalizable to all MSM populations or providers. Our sample of providers may be biased due to the heavy recruitment of providers working with or known by researchers affiliated with the participating sites. We also utilized different (but comparable) data collection modalities for provider interviews [41]. We did not have the opportunity to conduct a rigorous member check (informant feedback) for validation. Despite these limitations, these data provide insights about where current practices stand and whether and how new prevention options might fit into these geographically, politically and socially disparate locales across the Americas.

## Conclusion

In the US, MSM most in need of added HIV prevention were most poised to uptake new strategies. Since these interviews were conducted, PrEP has become widely available in New York and San Francisco. In addition to several PrEP demonstration projects underway to evaluate PrEP uptake and delivery, research has noted that interest and uptake among MSM is high when PrEP is offered as part of a comprehensive prevention package in STD clinics and a community health center [42]. Additionally, HIV self-test kits are already available over the counter in the US, though limited data on update would indicate that sales are modest at best, potentially due to the expense [43]. In the US, the crucial question for implementation of combination prevention strategies is now where to target resources; we believe efforts should focus on the non- and inconsistent-condom users. In Rio, MSM are poised to try a full complement of prevention options; the challenge lies with forging provider support and addressing potential structural barriers in implementing new technologies. Recent developments are encouraging, as the Brazilian government has implemented universal access to ARVs and is now supporting a PrEP demonstration project as well as collaborating on a home testing project supported by the US Centers for Disease Control; both protocols will address key implementation issues within the public health system. In Peru provision of new technologies could take longer to implement given the country’s more restrictive and centralized services for testing and treatment and slower scale up of ARV access. That said, the very recent adoption of expanded treatment guidelines (including eligibility at CD4 ≤ 500) and new Global Fund program aiming to expand testing into community-based settings could indicate a new era for prevention with MSM. Once ARV scale-up is implemented in Peru, discussion about new technologies could gain momentum; uptake, however, may be slow given provider misgivings and the lukewarm reactions of study participants. Men in Lima appear to be progressing towards a more accepting culture and may benefit initially from more intensive facilitation to improve discussion on the topic of sexual health with sex partners, focusing on increased testing and counseling options—prevention tools with which they are most comfortable and which could become available in community venues. If prevention conversations gain momentum and nuance, MSM in Peru could move towards a shift in thinking about using technologies such as PrEP and home testing; and, as in Brazil, this would require provider support and supportive policy.

Developing integrated prevention strategies requires careful attention to perceived preferences and needs of targeted communities, provider opinion, and to the policy context and capacity of current programming in each country. Assumptions that innovations identified in research and even those approved for distribution will lead to uptake and use underestimates the role of community, culture, gate-keepers of these innovations (providers) and existing practices among potential adopters. Supportive public policies will fuel prevention, just as unsupportive policies or providers and lack of community awareness can dampen use of new prevention tools. Results from the present study suggest that simultaneously engaging prospective consumers and providers in refining and optimizing intervention approaches will be key to expanding new prevention approaches for MSM.
